# A Mild Dyssynchronous Contraction Pattern Detected by SPECT Myocardial Perfusion Imaging Predicts Super-Response to Cardiac Resynchronization Therapy

**DOI:** 10.3389/fcvm.2022.906467

**Published:** 2022-05-31

**Authors:** Xiao Hu, Zhiyong Qian, Fengwei Zou, Siyuan Xue, Xinwei Zhang, Yao Wang, Xiaofeng Hou, Weihua Zhou, Jiangang Zou

**Affiliations:** ^1^Department of Cardiology, The First Affiliated Hospital of Nanjing Medical University, Nanjing, China; ^2^Department of Cardiology, The Affiliated Huaian No. 1 People's Hospital of Nanjing Medical University, Huaian, China; ^3^Montefiore Medical Center, Bronx, NY, United States; ^4^College of Computing, Michigan Technological University, Houghton, MI, United States

**Keywords:** CRT, mild dyssynchronous, contraction pattern, SPECT, super-response

## Abstract

**Background:**

Using single photon emission computed tomography myocardial perfusion imaging (SPECT MPI) with phase analysis (PA), we aimed to identify the predictive value of a new contraction pattern in cardiac resynchronization therapy (CRT) response.

**Methods:**

Left ventricular mechanical dyssynchrony (LVMD) was evaluated using SPECT MPI with PA in non-ischemic dilated cardiomyopathy (DCM) patients with left bundle branch block (LBBB) indicated for CRT. CRT super-response was defined as LV ejection fraction (EF) ≥50% or an absolute increase of LVEF >15%. The LV contraction was categorized as the mild dyssynchronous pattern when the phase standard deviation (PSD) ≤ 40.3° and phase histogram bandwidth (PBW) ≤ 111.9°, otherwise it was defined as severe dyssynchronous pattern which was further characterized as U-shaped, heterogeneous or homogenous pattern.

**Results:**

The final cohort comprised 74 patients, including 32 (43.2%) in mild dyssynchronous group, 17 (23%) in U-shaped group, 19 (25.7%) in heterogeneous group, and 6 (8.1%) in homogenous group. The mild dyssynchronous group had lower PSD and PBW than U-shaped, heterogeneous, and homogenous groups (*P* < 0.0001). Compared to patients with the heterogeneous pattern, the odds ratios (ORs) with 95% confidence intervals (CIs) for CRT super-response were 10.182(2.43–42.663), 12.8(2.545–64.372), and 2.667(0.327–21.773) for patients with mild dyssynchronous, U-shaped, and homogenous pattern, respectively. After multivariable adjustment, mild dyssynchronous group remained associated with increased CRT super-response (adjusted OR 5.709, 95% CI 1.152–28.293). Kaplan-Meier curves showed that mild dyssynchronous group demonstrated a better long-term prognosis.

**Conclusions:**

The mild dyssynchronous pattern in patients with DCM is associated with an increased CRT super-response and better long-term prognosis.

## Introduction

Cardiac resynchronization therapy (CRT) has been proven to be an effective strategy for patients with heart failure (HF) and electrical dyssynchrony (1). Left bundle branch block (LBBB) alone is a powerful predictor for CRT response. Although promising effects have been reported, individual response to CRT varies with up to one-third of patients with HF having no response to CRT (2, 3). Although the main mechanism of benefit from CRT is the correction of cardiac dyssynchrony, the relationship between left ventricular (LV) electrical and mechanical dyssynchrony may have a discrepancy. Animal studies have shown that mechanical delay exceeds electrical delay in ventricular pacing conditions (4). Delgado et al. (5) demonstrated that severe baseline LV mechanical dyssynchrony (LVMD) assessed by speckle-tracking echocardiography (STE) may predict favorable long-term prognosis in ischemic HF patients treated with CRT. Previous studies have demonstrated U-shaped pattern was deemed to have a favorable response rate to CRT (6–8). Cardiac magnetic resonance (CMR) and three-dimensional (3D) STE are non-invasive approaches to evaluate LV mechanical patterns and to investigate the effect of relevant electromechanical characteristics on CRT responses (9–11). Using CMR, Jackson et al. (10) found that the U-shaped contraction pattern is strongly predictive for CRT super-response. However, longer imaging time and inability to scan patients with implanted devices, have limited the advance of CMR (12). Similarly, STE relies heavily on operator experience and lacks high reproducibility.

Phase analysis (PA) is a contemporary non-invasive method based on single-photon emission computed tomography myocardial perfusion imaging (SPECT MPI) and has been applied to investigate LVMD, latest-excited sites, and myocardial scar load with good reproducibility and reliability (13, 14). The 13-segmentation polar map based on the PA can display the mean phase angle, which exhibits systolic dyssynchrony and identifies the late contracting segments.

In our clinical practice, we found that CRT candidates with a new mild dyssynchronous pattern identified by SPECT frequently achieve super-response. This study aims to explore the different contraction patterns using PA technique and to investigate their predictive values on CRT super-response.

## Materials and Methods

### Study Population

Seventy-four patients with non-ischemic dilated cardiomyopathy (DCM) who received resting SPECT MPI were enrolled in this study at The First Affiliated Hospital of Nanjing Medical University from May 2014 to July 2020. Indications for CRT in the present study included (1) sinus rhythm; (2) LV ejection fraction (LVEF) ≤ 35%; (3) LBBB with QRS duration ≥130 ms; (4) New York Heart Association (NYHA) functional class II to IV; (5) at least 3 months of optimized medical therapy before CRT implantation. The exclusion criteria were: (1) patients with persistent atrial fibrillation; (2) upgrade to CRT in pacing-dependent patients. The study was approved by the institutional review board, and written informed consent was obtained from all patients.

### SPECT MPI and Phase Analysis

All the patients underwent resting SPECT MPI with the injection of 20–30 mCi of Tc-99 m sestamibi before CRT implantation. The SPECT was performed approximately 60 min after injection, and dual-headed camera (CardioMD, Philips Medical Systems) was used to acquire MPI images with a standard resting protocol. The parameters contained a 20% energy window around 140 keV, 180 orbit, 32 steps with 25 s per step, 8-bin gating, and 64 planar projections per gate. Image reorientation and reconstruction were achieved through Emory Reconstruction Toolbox (ERToolbox; Atlanta, GA).

**Figure 1:**
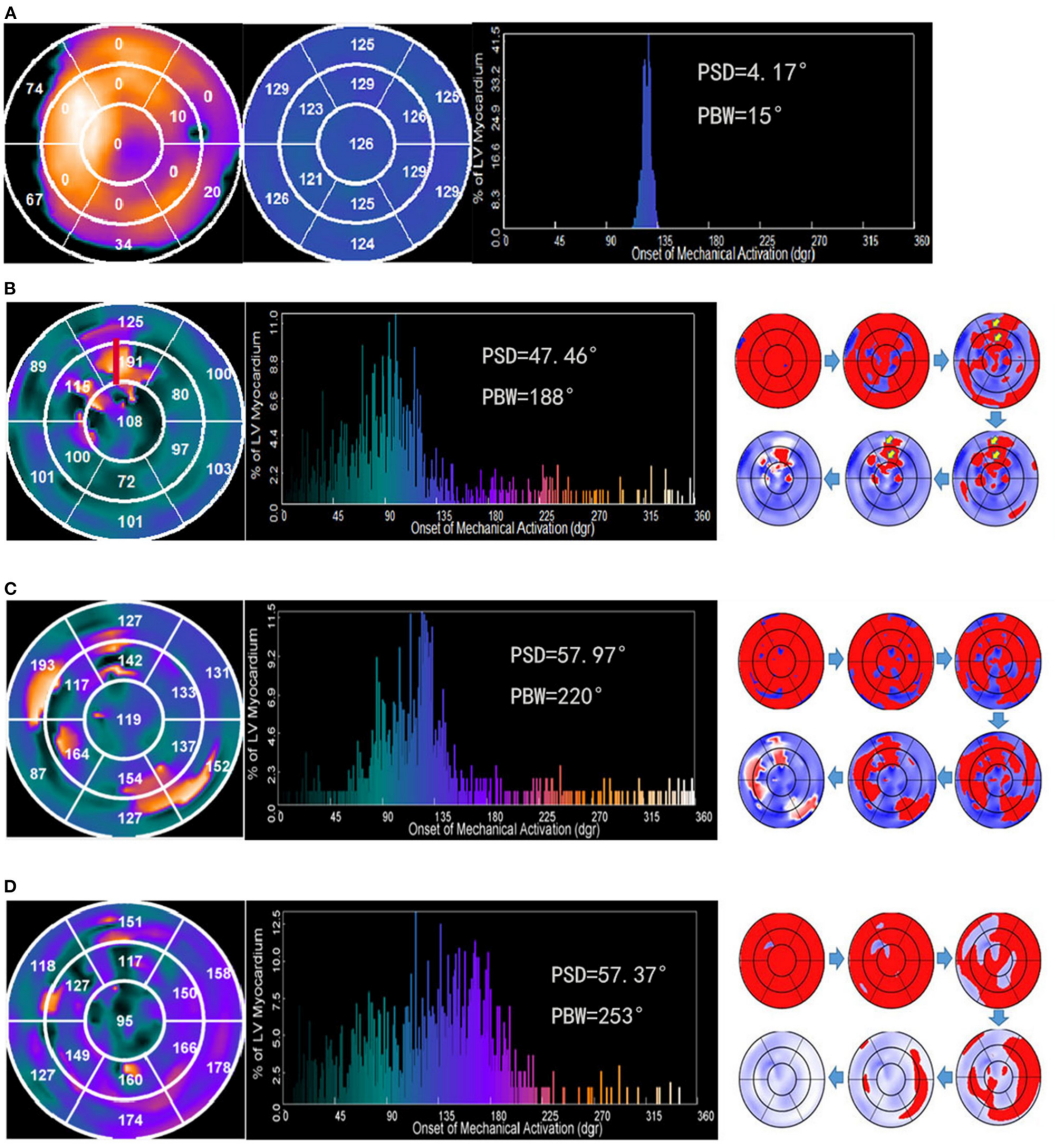
Examples illustrating four contraction patterns. Numbers in the regional contraction polar maps represents the mean phase angle in each segment. The big number or bright color represents the late contracting segments. **(A)** Polar map of regional myocardial viability (left). The bright color represents the viable myocardium. The perfusion with <50% of the maximum tracer uptake was defined as scar. The number indicates the percentage of scar in this segment. A patient with the mild dyssynchronous contraction pattern who had a super-response to CRT (middle). **(B)** A patient with the U-shaped contraction pattern who had a super-response to CRT. A line of block was apparent between the septum and lateral wall. The anterior wall was the latest contraction site (red). **(C)** A patient with the heterogeneous contraction pattern who did not have a super-response to CRT. The phase angles of each segment are incommensurable, which caused multiple sites with the significant contraction delays. **(D)** A patient with the homogeneous pattern who did not have a super-response to CRT. The propagation proceeded from the septum to lateral wall homogenously.

Using a 3D search for maximal count circumferential profiles in respective cardiac frame, LV sampling was obtained with an automatic algorithm (15). The images of SPECT MPI were used to quantify scarred myocardium. The percentage of tracer uptake was displayed on polar map using a 13-segmentation model (Figure 1A, left). LV sample with <50% of maximal myocardial uptake was defined as myocardial scar region, and scar burden was expressed as a percentage of myocardial scar over total LV myocardium. The 13-segmentation regional contraction polar maps displayed the mean phase angle, which exhibited the systolic dyssynchrony and identified the late contracting segments (Figure 1A, middle). The 1- harmonic Fourier approximation was applied to quantify the onset of LV mechanical contraction (14). Phase standard deviation (PSD) and phase histogram bandwidth (PBW) (Figure 1A, right) were used to assess global LVMD (13). The latest activated region had relatively large phases and appeared as a brighter region in the phase polar map. Moreover, wall thickening was used to assess the changes in maximal counts for a myocardial location throughout the cardiac cycle.

### Assessment of Contraction Patterns

A higher PSD or PBW was related to increased LVMD, and vice versa. Based on previous research (16), LVMD was defined as above the mean+2 standard deviations of PSD or PBW. A threshold of 40.3° for PSD and 111.9° for PBW were applied, which were derived from the control group of 8 LBBB patients with normal LV function. LVMD was considered to be present if at least one of the parameters was above the cutoff value. Two types of LV contraction patterns were demonstrated in our study. The LV contraction pattern was categorized as the mild dyssynchronous group when the PSD ≤ 40.3° and PBW ≤ 111.9° (Figure 1A). The severe dyssynchronous pattern was defined when the PSD >40.3° or PBW >111.9°. The severe dyssynchronous pattern included U-shaped pattern (Figure 1B) if a line of block was apparent in the direction of LV contraction propagation or heterogeneous pattern (Figure 1C) if multiple sites of significant contraction delays existed in the propagated direction of LV, or homogenous pattern (Figure 1D) if propagation proceeded from the septum to lateral wall homogenously. The contraction patterns were analyzed by two independent investigators. Consensus was reached by a third investigator for any disagreement.

### Echocardiographic Evaluation and Electrocardiographic Feature Analysis

All patients received transthoracic echocardiography by an ultrasound specialist who was blinded to the study at baseline and follow-up. LV end-systolic diameter (LVESD), LV end-diastolic diameter (LVEDD), and LVEF were recorded using the 2-dimensional modified biplane Simpson method. The ECG feature at baseline was analyzed. The patients were classified as q-SLBBB and V5&V6S group according to our previous study (17).

### CRT Implantation

The devices were implanted based on the standard procedures (18). The atrial lead was implanted in the right atrial appendage, right ventricular (RV) lead was placed at the RV apex or septum, and LV lead was implanted by targeting the posterolateral, lateral, anterolateral, or posterior coronary sinus side branch.

### CRT Super-Response and Clinical Outcomes

Patients were followed up 1, 3, and 6 months after CRT implantation. After 6 months, the patients were followed up at least once every year. CRT response was defined as an absolute increase of LVEF ≥5% at 6-month follow-up. CRT super-response was defined as LVEF ≥50% or an absolute increase of LVEF >15% at 6-month follow-up (19, 20). All-cause mortality or HF hospitalization was considered as the combined end point.

### Statistical Analysis

Categorical variables were expressed as numbers (n) or percentage (%). Continuous variables were expressed as mean ± standard deviation (SD) or medians (25–75th percentile) based on their normality following Kolmogorov–Smirnov test. Comparisons between groups were performed by the chi-square test or Fisher test for categorical variables, Student's *t*-test for parametric variable, and Mann Whitney U test for non-parametric variables. To protect against Type I error, Bonferroni's correction was used. Continuous data between multiple groups were compared using one-way ANOVA test followed by *post-hoc* test (least significant difference). Intergroup comparisons were analyzed using paired *t*-test. The stepwise logistic regression (*P* < 0.05 in the univariate regression were included and variables with *P* >0.05 in the multivariate regression were excluded) were performed to assess the independent predictors of CRT super-response. The Kaplan-Meier curve analysis was used to compare the all-cause mortality or hospitalization for HF. *P* < 0.05 was considered statistically significant. Statistical analysis was performed with SPSS (version 26, Statistical Package for the Social Sciences, International Business Machines, Inc., Armonk, NY, USA).

## Results

### Patient Characteristics

The baseline characteristics of the enrolled patients are summarized in Table 1. Sixty-six patients (89.2%) had IA indication for CRT implantation with QRS duration ≥150 ms. All the patients received available optimized medical therapy pre and post CRT. There was no statistical difference in the use of Beta -blockers, angiotensin converting enzyme inhibitors (ACEI)/angiotensin receptor blockers (ARB), and aldosterone antagonists or diuretics between groups. No significant difference was observed in the use of CRT-P or CRT-D device between groups. The majority of LV leads were positioned in either lateral or posterolateral branches of coronary veins. There was no statistically significant difference in LV lead positions between groups. Compared with the severe dyssynchronous group, mild dyssynchronous group had significantly higher LVEF and smaller LVEDD (28.66% ± 5.06 vs. 25.76 ± 5.04%, *P* = 0.017; 68 mm ± 6.96 mm vs. 76.45 mm ± 9.87 mm, *P* < 0.001, respectively). PSD and PBW were also significantly higher in the severe dyssynchronous group than in the mild dyssynchronous group (57.93 ± 12.38° vs. 22.2 ± 7.18°, *P* < 0.0001; 205.71 ± 54.64° vs. 69.84° ± 21°, *P* < 0.0001, respectively).

**Table 1:** Baseline characteristics of 74 patients with LBBB.

**Variables**	**All** **(*n* = 74)**	**Mild dyssynchronous** **(*n* = 32)**	**U-shaped** **(*n* = 17)**	**Heterogeneous** **(*n* = 19)**	**Homogenous** **(*n* = 6)**	***P***
Age (year)	66.11 ± 11.07	65.22 ± 11.19	68.35 ± 11.06	66.95 ± 11.86	61.83 ± 8.23	0.60
Male (%)	55 (74.3%)	25 (78.1%)	12 (70.6%)	15 (78.9%)	3 (50%)	0.49
Hypertension (%)	30 (40.5%)	15 (46.9%)	6 (35.3%)	6 (31.6%)	3 (50%)	0.66
Diabetes (%)	13 (17.6%)	4 (12.5%)	1 (5.9%)	7 (36.8%)	1 (16.7%)	0.07
Renal dysfunction (%)	3 (4.1%)	2 (6.3%)	1 (5.9%)	0 (0%)	0 (%)	0.66
Smoking (%)	27 (36.5%)	12 (37.5%)	5 (29.4%)	8 (42.1%)	2 (33.3%)	0.88
NT-proBNP (ng/L)	1,968 (1,166–4225.8)	1,505 (897.5–3919.8)	1,927 (1,164–4,163)	2,915 (1670–5571)	2,422 (827–5,280)	0.63
NYHA II/ III /IV	21/40/13	11/17/4	2/9/6	6/11/2	2/3/1	0.39
QRSd (ms)	177 (162.8–188)	175 (160.5–187.5)	172 (160–188.5)	180 (170–188)	173.5 (165–193)	0.78
ACEI/ARB (%)	62 (83.8%)	25 (78.1%)	16 (94.1%)	15 (78.9%)	6 (100%)	0.31
Beta-blocker (%)	68 (91.9%)	28 (87.5%)	16 (94.1%)	18 (94.7)	6 (100%)	0.64
Aldosterone antagonist (%)	66 (89.2%)	29 (90.6%)	13 (76.5%)	18 (94.7%)	6 (100%)	0.24
Diuretics (%)	68 (91.9%)	29 (90.6%)	15 (88.2%)	18 (94.7%)	6 (100%)	0.78
CRT-D (%)	41 (55.5%)	19 (59.4%)	7 (41.2%)	12 (63.2%)	3 (50%)	0.55
**ECG pattern**						
q-SLBBB	1 (1.3%)	1 (3.1%)	0 (0%)	0 (0%)	0 (0%)	0.68
V5&V6 S	21 (22.8%)	7 (21.9%)	2 (11.8%)	6 (31.6%)	4 (66.7%)	0.06
**Echocardiogram**						
LVEF (%)	27.8 (23.48–30.83)	30.2 (25.4–31.98)	29.2 (24.3–31.75)	23.5 (20.2–27.6)	25.2 (22.48–30.4)	**0.01**
LVEDD (mm)	71.5 (67–80)	68 (63.3–72.8)	72 (67–77.5)	82 (71–88)	78.5 (68.25–83.5)	**<0.01**
LVESD (mm)	61.5 (57–71.25)	59 (56.25–63)	61 (56.5–68.5)	74 (62–79)	69 (57.75–74.5)	**<0.01**
LAD (mm)	46 (41–51)	44 (40–48.75)	46 (43–49)	52 (46–56)	45 (42.5–51.5)	**0.02**
Moderate/ severe MR (%)	59 (79.7%)	24 (75%)	15 (88.2%)	14 (73.7)	6 (100%)	0.37
Moderate/ severe TR (%)	25 (33.8%)	9 (28.1%)	7 (41.2%)	7 (36.8%)	2 (33.3%)	0.81
**SPECT MPI**						
PSD, degree	43.2 (23.73–58.31)	23.06 (17.24–26.6)	52.98 (47.95–56.92)	64.64 (57.97–74.45)	46.25 (40.77–61.93)	**<0.01**
PBW, degree	137 (73–212.25)	70.5 (58.25–83.75)	188 (154.5–225)	241 (209–279)	164 (131.5–208.25)	**<0.01**
Scar burden (%)	29.42 (22.66–39.74)	24.29 (16.92–36.95)	27.26 (23.19–32.67)	37.07 (30.09–43.55)	38.82 (29.75–48.66)	**0.01**
Wall thickening (%)	32.48 (15.11–44.27)	14.03 (9.23–20.91)	40.82 (34.06–49.18)	44.5 (33.35–53.09)	38.34 (27.74–48.37)	**<0.01**
**LV lead position**						0.25
Anterolateral	10 (13.5%)	2 (6.3%)	2 (11.8%)	4 (21.1%)	0	
Lateral	30 (40.5%)	13 (40.6%)	7 (41.2%)	9 (47.4%)	3 (50%)	
Posterolateral	29 (39.2%)	14 (43.8%)	8 (47%)	4 (21.1%)	3 (50%)	
Posterior	5 (6.8%)	3 (9.4%)	0 (0%)	2 (10.5%)	0 (0%)	

*Values are presented as n (%) or mean ± SD or, median (interquartile range). Bold values are statistically significant*.

*NYHA, New York Heart Association; ACEI, angiotensin-converting enzyme inhibitor; ARB, angiotensin II receptor blocker; NT-proBNP, N-terminal pro-brain natriuretic peptide; q-SLBBB, Patients who met typical LBBB criteria along with q waves in leads I, V5, and V6; V5&V6S, Patients with absence of q waves in leads I, V5, and V6 and S wave in leads V5 and V6; LVEDD, left ventricular end-diastolic diameter; LVESD, left ventricular end-systolic diameter; LAD, left atrial diameter; LVEF, left ventricular ejection fraction; PSD, phase standard deviation; PBW, phase histogram bandwidth; MR, mitral regurgitation; TR, tricuspid regurgitation*.

**Figure 2:**
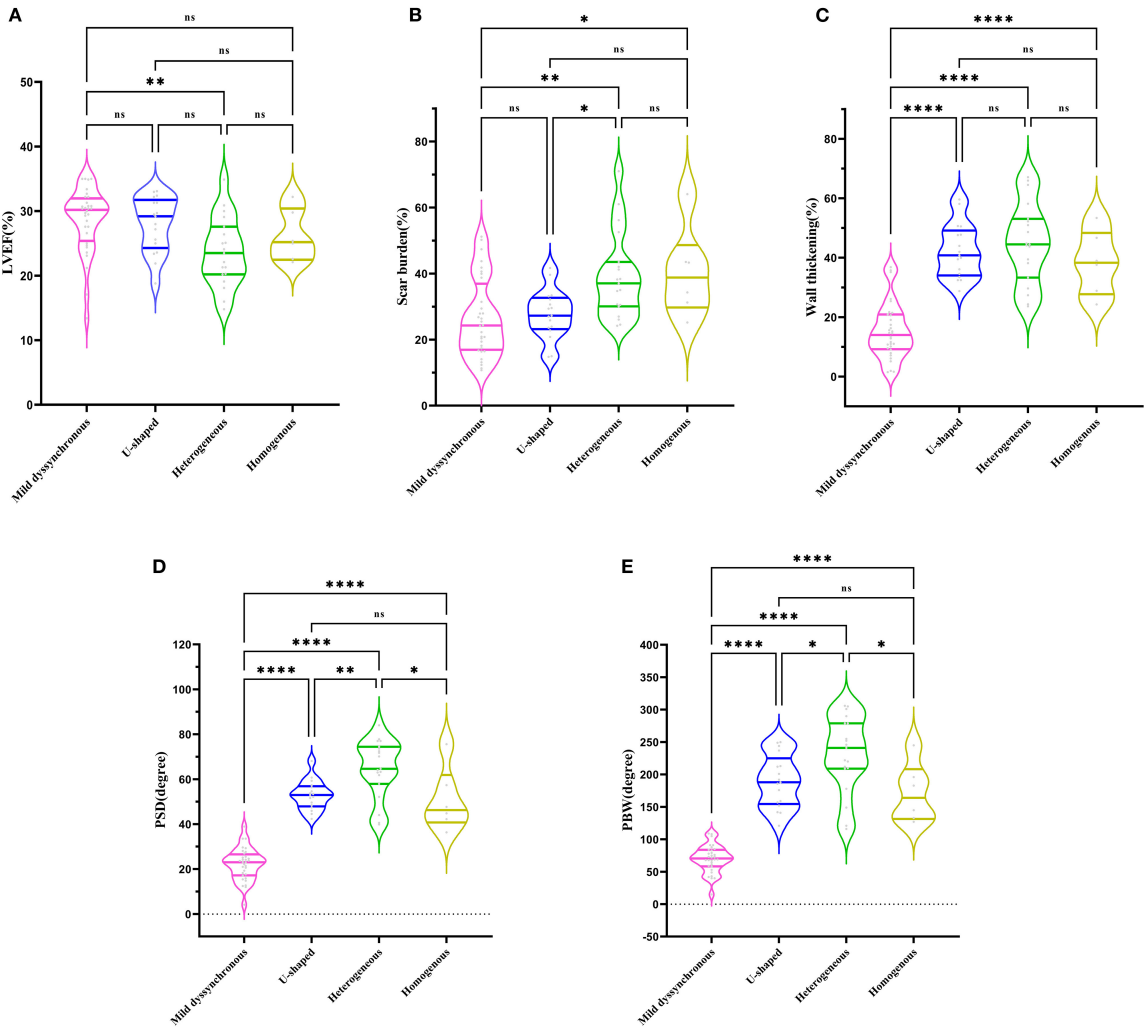
Comparison of LVEF, scar burden, wall thickening, PSD, and PBW in different groups. **(A)** Mild dyssynchronous group had a similar LVEF with U-shaped and homogenous group, significantly higher than heterogeneous group. **(B)** Mild dyssynchronous group had a similar scar burden with U-shaped group, significantly lower than heterogeneous group and homogenous group. **(C)** Mild dyssynchronous group had a lower wall thickening than U-shaped, heterogeneous, and homogenous group. **(D)** Mild dyssynchronous group had a lower PSD than U-shaped, heterogeneous, and homogenous group. **(E)** Mild dyssynchronous group had a lower PBW than U-shaped, heterogeneous, and homogenous group. *P < 0.05; **P < 0.01; ****P < 0.0001. ns, no statistically significant.

Based on the contraction pattern, the severe dyssynchronous group was further trichotomized into three groups: U-shaped (*n* = 17), heterogeneous (*n* = 19), and homogenous (*n* = 6). Mild dyssynchronous group had a similar LVEF with U-shaped and homogenous group, significantly higher than heterogeneous group (*P* < 0.01) (Figure 2A). Mild dyssynchronous group had a similar scar burden with U-shaped group, significantly lower than heterogeneous group and homogenous group (*P* < 0.05) (Figure 2B). Wall thickening was lower in mild dyssynchronous group than U-shaped, heterogeneous, and homogenous groups (*P* < 0.0001), whereas it did not reach significant difference among the severe dyssynchronous groups (Figure 2C). U-shaped group had higher PSD and PBW than mild dyssynchronous group (*P* < 0.0001), but lower than heterogeneous group (*P* < 0.05). Heterogeneous and homogenous groups had significantly higher PSD and PBW than mild dyssynchronous group (*P* < 0.0001) (Figures 2D,E).

### Echocardiographic Measurements at Follow-Up

#### LVEF and LVEDD Changes

**Figure 3:**
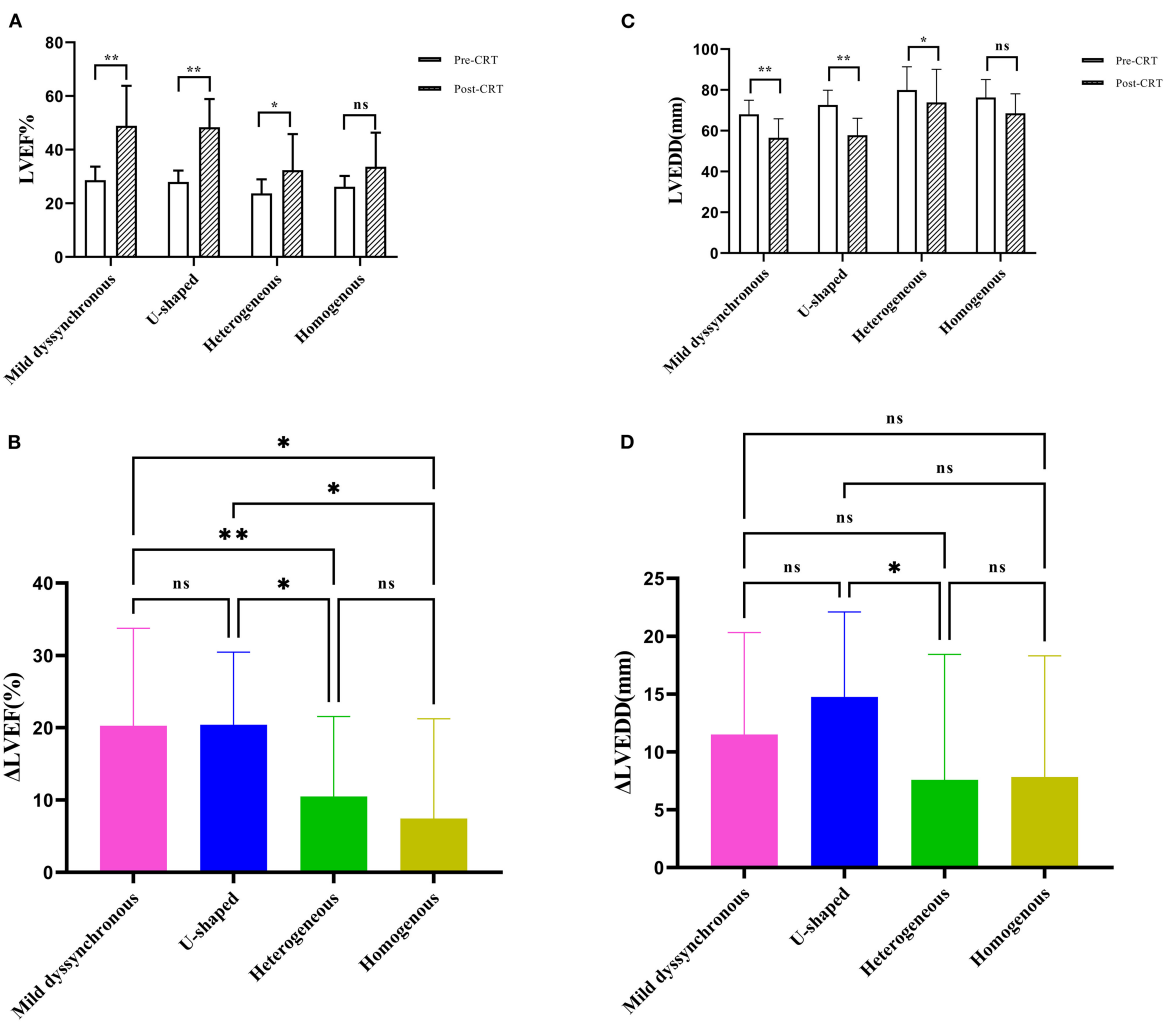
Comparison of echocardiographic measurements. **(A)** The panel shows the comparison of LVEF between baseline (pre-CRT) and post-CRT. **(B)** The panel shows the comparison of ΔLVEF among the four groups. **(C)** The panel shows the comparison of LVEDD between pre-CRT and post-CRT. **(D)** The panel shows the comparison of ΔLVEDD among the four groups. *P < 0.05; **P < 0.01; ns, no statistically significant. ΔLVEF, the changes of left ventricular ejection fraction; ΔLVEDD, the changes of left ventricular end-diastolic diameter.

The LVEF in each group was significantly increased at follow-up except the homogeneous group (mild dyssynchronous: 28.66 ± 5.06% vs. 48.91 ± 14.9%, *P* < 0.01; U-shaped: 27.95 ± 4.29% vs. 48.39 ± 10.51%, *P* < 0.01; heterogeneous: 23.67 ± 5.28% vs. 32.37 ± 13.48%, *P* < 0.05) (Figure 3A). Among these 4 groups, the change in LVEF showed significant differences, especially between the mild dyssynchronous group and the heterogeneous group (*P* < 0.01) (Figure 3B).

The LVEDD in each group was significantly decreased compared with baseline except the homogeneous group (mild dyssynchronous: 68 ± 6.96 mm vs. 56.5 ± 9.38 mm, *P* < 0.01; U-shaped: 72.59 ± 7.25 mm vs. 57.82 ± 8.29 mm, *P* < 0.01; heterogeneous: 79.95 ± 11.46 mm vs. 73.84 ± 16.26 mm, P < 0.05) (Figure 3C). The change in LVEDD showed significant difference only between the U-shaped group and the heterogeneous group (*P* < 0.05) (Figure 3D).

#### CRT Super-Response Rate

**Figure 4:**
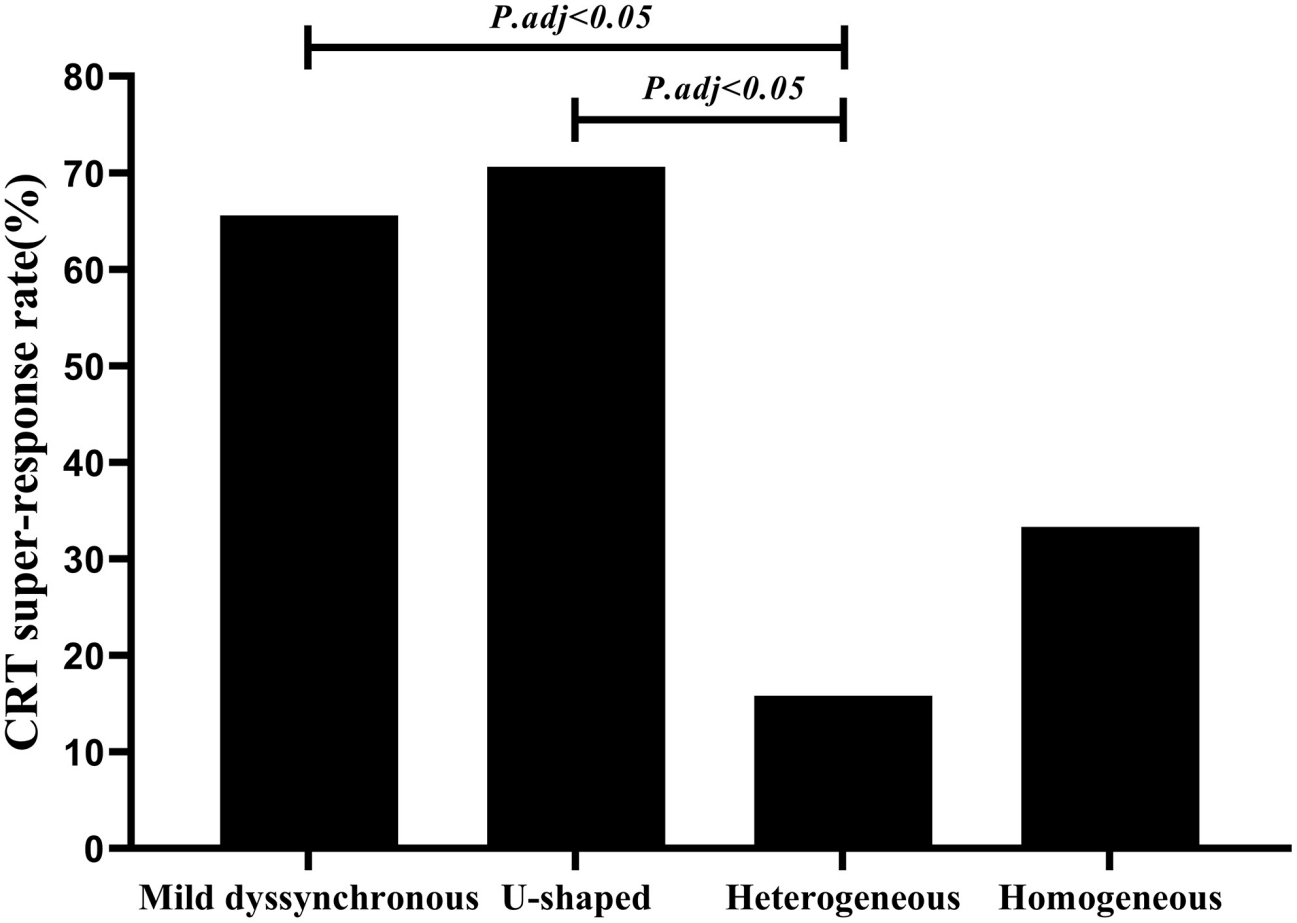
Comparison of CRT super-response rate among four groups. Mild dyssynchronous and U-shaped groups had significantly higher CRT super-response rates than the heterogeneous group (*P*.adj < 0.05). adj, adjusted. Multiple comparisons was adjusted by Bonferroni's correction.

At the 6-month follow-up, 50 patients (67.6%) achieved CRT response, and 38 patients (51.4%) achieved CRT super-response. CRT super-responders accounted for a large percentage of patients in the mild dyssynchronous group and U-shaped group (65.6 vs. 70.6%, *P* = 0.72), only 15.8% in the heterogeneous group and 33.3% in the homogenous group. The mild dyssynchronous and U-shaped groups had significantly higher CRT super-response rates than the heterogeneous group (Figure 4).

### Predictive Value of Contraction Pattern

In univariate analysis, compared with the heterogeneous pattern, the odds ratios (ORs) with 95% confidence intervals (CIs) for CRT super-response were 10.182 (2.43–42.663), 12.8 (2.545–64.372), and 2.667 (0.327–21.773) for the mild dyssynchronous, U-shaped, and homogenous group, respectively. PSD and scar burden analyzed as continuous variables were associated with poor benefit from CRT [OR 0.584 per 1 standard deviation (SD) increase, 95% CI 0.358–0.951; *P* = 0.031; OR 0.411 per 1 SD increase, 95% CI 0.228–0.74; *P* = 0.003; respectively]. After multivariable adjustment with V5&V6 S, LVEF, and scar burden, mild dyssynchronous and U-shaped groups remained associated with increased CRT super-response compared to heterogeneous group (adjusted OR 5.709, 95% CI 1.152-28.293; *P* = 0.033; adjusted OR 6.81, 95% CI 1.198–38.718; *P* = 0.031, respectively) (Table 2).

**Table 2:** Univariate analysis and multivariable models for CRT super-response.

**Characteristics**	**Univariate analysis**		**Multivariate analysis**	
	**OR (95% CI)**	***P***	**OR (95% CI)**	***P***
Age (per 10year)	1.05 (0.693–1.59)	0.818		
Male (%)	0.701 (0.245–2.01)	0.509		
Diabetes (%)	0.353 (0.098–1.271)	0.111		
Hypertension (%)	0.913 (0.361–2.311)	0.848		
Smoke (%)	1.032 (0.4–2.661)	0.948		
Alcohol user (%)	1.786 (0.574–5.559)	0.317		
Renal dysfunction (%)	1.944 (0.169–22.423)	0.594		
NT-proBNP (per 100 ng/L)	0.998 (0.986–1.009)	0.701		
QRSd (per 10 ms)	1.25 (0.938–1.666)	0.127		
**NYHA (%)**				
II	Ref.			
III	0.995 (0.346–2.866)	0.993		
IV	2.475 (0.577–10.617)	0.223		
LVEF (%)	1.152 (1.040–1.275)	**0.007**	1.066 (0.947–1.201)	0.290
V5&V6 S	0.238 (0.075–0.756)	**0.015**	0.292 (0.076–1.119)	0.073
**LV lead position**				
Lateral vs. Anterolateral	0.882 (0.187–4.158)	0.874		
Posterolateral vs. Anterolateral	1.417 (0.295–6.814)	0.664		
Posterior vs. Anterolateral	0.667 (0.069–6.409)	0.725		
Posterolateral vs. Lateral	1.606 (0.582–4.426)	0.360		
Posterior vs. Lateral	0.756 (0.111–5.149)	0.775		
Posterior vs. Posterolateral	0.471 (0.068–3.261)	0.445		
ACEI/ARB (%)	0.714 (0.204–2.496)	0.598		
Beta-blocker (%)	0.189 (0.021–1.7)	0.137		
PSD° (Per 1 SD)	0.584 (0.358–0.951)	**0.031**		
PBW° (Per 1 SD)	0.665 (0.413–1.07)	0.093		
Scar burden% (Per 1 SD)	0.411 (0.228–0.74)	**0.003**	0.661 (0.333–1.313)	0.237
Mild dyssynchronous vs. U-shaped	0.795 (0.223–2.841)	0.725		
Mild dyssynchronous vs. Heterogeneous	10.182 (2.43–42.663)	**0.002**	5.709 (1.152–28.293)	**0.033**
Mild dyssynchronous vs. Homogenous	3.818 (0.602–24.222)	0.155		
U-shaped vs. Heterogeneous	12.8 (2.545–64.372)	**0.002**	6.81 (1.198–38.718)	**0.031**
U-shaped vs. Homogenous	4.8 (0.655–35.198)	0.123		
Homogenous vs. Heterogeneous	2.667 (0.327–21.733)	0.36		

*CI, confidence interval; OR, odds ratio; Ref, reference; SD, standard deviation; other abbreviations as in Table 1. Bold values are statistically significant*.

### Long-Term Clinical Outcome

**Figure 5:**
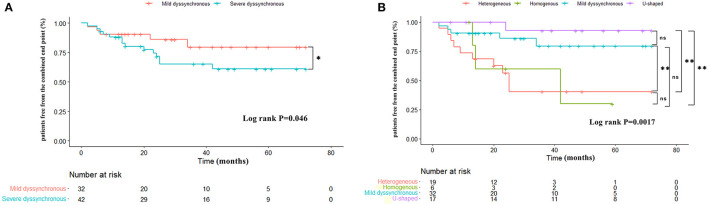
Kaplan-Meier curves comparing survival free of the combined end point in different groups. **(A)** The mild dyssynchronous group demonstrated a better long-term prognosis than the severe dyssynchronous group. **(B)** The incidence of HF hospitalization or all-cause mortality is significantly lower in the mild dyssynchronous group than in the heterogeneous group, which was similar to the U-shaped group. Log-rank *P*: *P < 0.05, **P < 0.01. ns, no statistically significant.

Over a mean follow-up of 37.48 ± 23.07 months, 19 patients (25.7%) reached the combined end point of HF hospitalization or all-cause mortality, 5 (15.6%) in the mild dyssynchronous group, 1 (5.9%) in the U-shaped group, 10 (52.6%) in the heterogeneous group, and 3 (50%) in the homogenous group. The mild dyssynchronous group demonstrated a better long-term prognosis than the severe dyssynchronous group (log-rank *P* = 0.046) (Figure 5A). The incidence of HF hospitalization or all-cause mortality was significantly lower in the mild dyssynchronous group than in the heterogeneous group (*P* < 0.01), which was similar to the U-shaped group. There is no difference in the incidence of HF hospitalization or all-cause mortality between patients with the heterogeneous pattern and the homogenous pattern (*P* > 0.05). Kaplan-Meier curves for the 4 groups are presented in Figure 5B.

## Discussion

The major findings of this study are: (1) The mild dyssynchronous pattern was observed in 43.2% of patients with DCM in our cohort; (2) The mild dyssynchronous pattern is associated with a favorable CRT super-response comparable with the U-shaped pattern, however the heterogeneous pattern showed a lesser response to CRT; (3) understanding contraction patterns in patients with LBBB provides additional predictive value for CRT super-response.

### Contraction Patterns and CRT Efficacy

Our study investigated a new mild dyssynchronous contraction pattern and its association with CRT super-response. These patients achieved favorable outcomes which led us to explore this new contraction pattern in patients with LBBB. In this study, 32 patients (43.2%) were identified by SPECT to have a mild dyssynchronous contraction pattern. The phase angle of each segment was comparable without obvious early or late propagation in adjacent segments. To compare the dyssynchrony of different contraction patterns, we also calculated the deviations between the maximum and minimum phase angles of different segments in each contracting pattern. Mild dyssynchronous group had the least mean deviations of phase angles compared to U-shaped group, heterogeneous group, and homogeneous group (37.53 vs. 90.29, 80.95, and 81.33°, *P* < 0.001).

Previous studies using non-contact mapping (NCM), STE, and CMR have identified two major contraction patterns (7, 9, 10, 21–23). The U-shaped pattern is characterized by a U-shaped contraction propagation caused by a line of block. Fung et al. (9) reported that the block lines could exist in the anterior, septal, or inferior wall. The lines of block in our study were mainly observed in anterior wall (*n* = 11) and septal wall (*n* = 6). Previous studies revealed that U-shaped pattern was related to a favorable CRT response. Fung et al. (9) and Sohal et al. (23) demonstrated that the volumetric CRT response was 80% for the U-shaped pattern. Tao et al. (24) identified three distinct patterns: U-shaped, heterogeneous, and homogenous. They found that U-shaped contraction pattern was also associated with improved CRT response. Homogenous pattern was characterized by propagation proceeded from the septum to lateral wall homogenously, which was associated with a lesser response to CRT. It was also seen in our study, which was consistent with that in previous studies. Approximately 34.7, 49, and 51.9% of patients were identified as having the homogenous pattern in these studies (9, 10, 23). The relatively lower prevalence of the homogenous pattern in this study (8.1%) was likely due to different inclusion criteria. Our patients presented with LBBB and DCM. The significance of this group lies on further expansion of sample size in the follow-up research to clarify the issue.

However, no study reported the CRT response in patients with mild dyssynchronous contraction pattern characterized by SPECT. In the study by Boogers et al. (25), they also demonstrated low values of PSD and PBW with gated MPI SPECT (GMPS) to predict the response to CRT. Forty patients with heart failure (30% with DCM), an LVEF ≤ 35%, and a QRS ≥120 ms underwent GMPS were enrolled. Particularly, a cutoff value of 72.5° for PBW and 19.6° for PSD yielded a sensitivity of 83% and a specificity of 81% for prediction of response to CRT. The relatively low cutoff values may be related to differences in study populations or to differences in software packages. Our study is the first to show that this mild dyssynchronous contraction pattern was strongly associated with CRT super-response in patients with LBBB and non-ischemic DCM similar to the previously described U-shaped pattern.

In the mild dyssynchronous group, eight patients without LV dyssynchrony were identified based on a threshold of 24.4° for PSD and 62.3° for PBW, which were derived from the healthy people with normal LV function (13). This group had nearly normal PSD and PBW (13.09 ± 4.03°; 42.5 ± 12.88°, respectively). They had significantly less scar burden, smaller LVEDD, and left atrial diameter (LAD). Similarly, they had short duration of HF history. This group with LV synchronous pattern also exhibited a super-response rate of 50%.

### Similar Super-Response Rate Between Mild Dyssynchronous and U-Shaped Groups

Previous studies showed that CRT response was associated with the presence of baseline LV dyssynchrony measured by PA of GMPS (25–27). Henneman et al. (26) established a cutoff value of 135° for PBW and 43° for PSD for prediction of CRT response. Similar studies also showed significantly larger PSD and PHB values in CRT responders (25, 27). However, the accuracy of GMPS to predict response to CRT was not perfect. Actually, it is not realistic to predict the accuracy of CRT response based solely on LV dyssynchrony. Recent work has indicated that scar tissue was closely related to LV dyssynchrony (28). Myocardial scar hampered with the propagation of mechanical activation and contributed to more dyssynchronous contraction. It has been shown that the extent of scar tissue was negatively correlated with CRT response (29). It could be assumed that if baseline LV dyssynchrony is mainly caused by scar, patients with HF would not have a better response to CRT. Hung et al. (30) demonstrated that the global LV dyssynchrony could not predict CRT response, only LV dyssynchrony in the viable myocardium had a better response to CRT. Our results are in line with earlier work by Wang et al. (31). They found systolic PSD, systolic PBW, diastolic PSD, and diastolic PBW in the CRT response group were statistically significant lower than those in the non-response group (*P* < 0.05). Meanwhile, the CRT non-response group showed more scar burden, and more LV leads were located in the scarred segments. In our clinical practice, we found that CRT candidates with a mild dyssynchronous pattern which was presented in this study frequently achieve super-response. The mild dyssynchronous group had less scar burden, and we speculated that the less scar burden may contributed to the lower PSD and PBW, which led to a favorable response to CRT.

In our cohort, we found a significant relationship between the contraction pattern and LV function. The mild dyssynchronous group had the lowest LAD, LVEDD, PSD, and PBW. Moreover, these individuals had a significantly higher EF than those with the heterogeneous pattern, which suggests that worse LV function could be underlying the difference in contraction patterns. It has also been demonstrated that wall thickening correlates well with LVEF (32). Mild dyssynchronous group had a lower wall thickening than the other 3 groups, indicating that the abnormal wall thickening plays a significant role in LV remodeling. The MADIT-CRT study (33) showed that smaller baseline LA volume index had a high predictive value to identify CRT super-responders. Consistent with this finding, we also found the mild dyssynchronous group had smaller LAD. This may contributed to the favorable super-response to CRT. Clinical studies reported that patients with smaller baseline LVEDD were more likely to be CRT super-responders (34, 35). The size of LV reflects the stage of structural remodeling in HF patients. Apoptosis and fibrosis have been identified as early features of HF. The pathogenesis of HF is associated with progressive inherent dysfunction, degeneration and loss of viable cardiomyocyte (36). Therefore, among HF patients with larger LVEDD, the loss of cardiomyocyte and subsequent fibrosis must be much more severe and extensive. It is easy to understand that a heart without enough viable cardiomyocyte is unlikely to respond to CRT.

Another possible explanation for high CRT super-response in the mild dyssynchronous group may be the presence of less scar tissue near the LV pacing lead. The presence, position, and burden of myocardial scar have been reported to affect CRT response (37). Linear regression analysis also showed that the baseline scar burden was negatively correlated to the changes of LVEF after CRT (*P* < 0.001) (Supplementary Figure S1). Zhang et al. (38) reported the extent of scarred LV segments identified on SPECT was negatively correlated with CRT response. Our results showed that the mild dyssynchronous contraction pattern was associated with less scar burden compared to the heterogeneous pattern. Our 8 patients with LBBB and normal LV function serving as the control group also had less scar burden than the mild dyssynchronous group (8.73 ± 3.28% vs. 26.52 ± 11.85%, *P* < 0.0001). This further demonstrated that patients with less scar burden were more likely to be super-responders.

Moreover, the mild dyssynchronous group had fewer patients with V5&V6S ECG phenotypes compared with the heterogeneous group. Our previous study found that an S wave in lead V6 predicts poor response to CRT (17).

### Possible Mechanism of the Mild Dyssynchronous Pattern

DCM is an extremely heterogeneous disease presented with mild or moderate LV dysfunction (39). Electrical activation propagates from RV to LV through the intraventricular septum in patients with LBBB. The subsequent dyssynchronous contraction and relaxation lead to dilated LV and eventually HF. It is currently unclear whether each contraction pattern truly represents their different electrical activation. We speculated that the mild dyssynchronous pattern may be the early stage of HF, and the primary etiology for a substantial of patients may be the bundle branch block. Correction of LBBB by biventricular pacing could lead to electrical and mechanical resynchrony and normalization of LV function. A recent study showed that using left bundle branch pacing to correct LBBB can cure DCM patients, which could be inferred that LBBB played a vital role in occurrence of DCM. The hypothesis of LBBB-induced DCM seems to be a reasonable conception (40). Without prompt intervention, LV continues to dilate and its contraction sequence becomes more complex leading to different contraction morphologies such as the heterogeneous, homogenous, and U-shaped patterns identified in this study. Further work is needed to investigate each contraction pattern.

### Limitations

First, this was a retrospective observational study with small sample size. Second, except for LVMD assessed by SPECT, no electrical activation parameters were collected. Third, our classification of mechanical contraction pattern has not been used in other studies and thus needs more validation. Prospective multicenter studies in larger cohorts are warranted.

## Conclusion

The mild dyssynchronous pattern assessed by SPECT is associated with increased CRT super-response and better long-term prognosis in patients with DCM and LBBB, which was comparable with the U-shaped pattern. This finding may have significant clinical significance in selecting proper CRT patients and further investigation is needed to validate its clinical application.

## Data Availability Statement

The raw data supporting the conclusions of this article will be made available by the authors, without undue reservation.

## Ethics Statement

The studies involving human participants were reviewed and approved by Institutional Review Board of Nanjing Medical University. The patients/participants provided their written informed consent to participate in this study.

## Author Contributions

JZ conceived the study. ZQ and FZ performed image interpretation. SX collected the data. YW and XZ analyzed the data. XHu wrote the first draft of the manuscript. XHo and WZ edited the manuscript. All authors discussed the results and commented on the manuscript.

## Funding

This study was supported by National Natural Science Foundation of China (No: 82070521, PI: JZ).

## Conflict of Interest

The authors declare that the research was conducted in the absence of any commercial or financial relationships that could be construed as a potential conflict of interest.

## Publisher's Note

All claims expressed in this article are solely those of the authors and do not necessarily represent those of their affiliated organizations, or those of the publisher, the editors and the reviewers. Any product that may be evaluated in this article, or claim that may be made by its manufacturer, is not guaranteed or endorsed by the publisher.
