# Supplementary material for: A Mild Dyssynchronous Contraction Pattern Detected by SPECT Myocardial Perfusion Imaging Predicts Super-Response to Cardiac Resynchronization Therapy
Source: Front Cardiovasc Med. 2022 May 31;9:906467. doi: 10.3389/fcvm.2022.906467 (PMC9194389; doi:10.3389/fcvm.2022.906467)
Supplement: Supplementary file 1 [file Data_Sheet_1.DOCX]

**
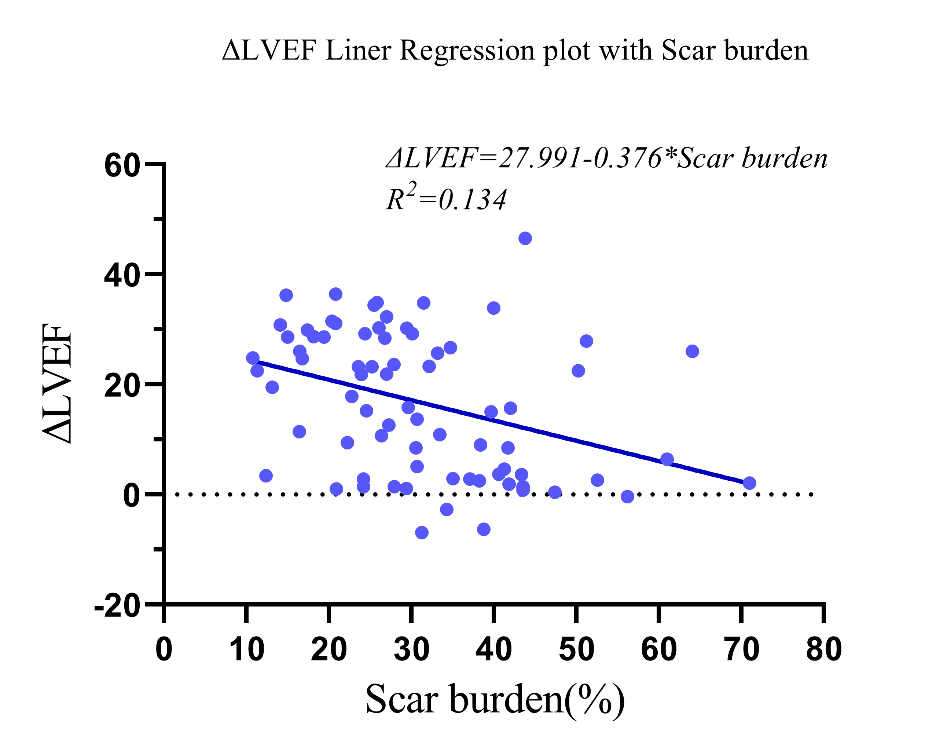
**

**Supplementary Figure 1.** Linear regression plotting of the changes of LVEF after CRT and baseline Scar burden. The baseline scar burden was negatively correlated to the changes of LVEF after CRT (P < 0.001)
